# Seroprevalence of Flavivirus Neutralizing Antibodies in Thailand by High-Throughput Neutralization Assay: Endemic Circulation of Zika Virus before 2012

**DOI:** 10.1128/mSphere.00339-21

**Published:** 2021-07-14

**Authors:** Atsushi Yamanaka, Mami Matsuda, Tamaki Okabayashi, Pannamthip Pitaksajjakul, Pongrama Ramasoota, Kyoko Saito, Masayoshi Fukasawa, Kentaro Hanada, Tomokazu Matsuura, Masamichi Muramatsu, Tatsuo Shioda, Ryosuke Suzuki

**Affiliations:** a Mahidol-Osaka Center for Infectious Diseases, Faculty of Tropical Medicine, Mahidol University, Bangkok, Thailand; b Mahidol-Osaka Center for Infectious Diseases, Research Institute for Microbial Diseases, Osaka University, Osaka, Japan; c Department of Virology II, National Institute of Infectious Diseases, Tokyo, Japan; d Department of Laboratory Medicine, The Jikei University School of Medicine, Tokyo, Japan; e Center for Animal Disease Control, University of Miyazaki, Miyazaki, Japan; f Department of Social and Environmental Medicine, Faculty of Tropical Medicine, Mahidol University, Bangkok, Thailand; g Center of Excellence for Antibody Research (CEAR), Faculty of Tropical Medicine, Mahidol University, Bangkok, Thailand; h Department of Biochemistry and Cell Biology, National Institute of Infectious Diseases, Tokyo, Japan; i Department of Viral Infections, Research Institute for Microbial Diseases, Osaka University, Osaka, Japan; j Department of Biological Science and Technology, Tokyo University of Science, Tokyo, Japan; Stanford University School of Medicine

**Keywords:** single-round infectious particles, flavivirus seroprevalence, neutralizing antibody, dengue virus, Zika virus

## Abstract

Thailand is a hyperendemic country for flavivirus infections in Southeast Asia. Although the reporting system for flavivirus surveillance in Thailand is well established, syndromic surveillance tends to underestimate the true epidemiological status of flaviviruses due to the majority of infections being asymptomatic. To accurately understand the prevalence of flaviviruses in endemic regions, we performed neutralization tests against multiple flaviviruses using 147 serum samples from healthy donors collected from four distinct regions in Thailand. Single-round infectious particles (SRIP) for six flaviviruses, dengue virus types 1 to 4 (DENV-1 to -4), Japanese encephalitis virus (JEV), and Zika virus (ZIKV), were used as antigens for developing a safe, high-throughput neutralization assay. Titers of neutralizing antibodies (NAbs) against the six flaviviruses revealed that DENV-1 and DENV-2, followed by ZIKV were the predominant circulating flaviviruses in a total of four regions, whereas the prevalence of NAbs against JEV varied among regions. Although the seroprevalence of ZIKV was low relative to that of DENV-1 and DENV-2, the findings strongly suggested that ZIKV has been circulating at a sustained level in Thailand since before 2012. These findings not only demonstrated the application of an SRIP-neutralization test in a serological study, but also elucidated the circulation and distribution trends of different flaviviruses in Thailand.

**IMPORTANCE** Neutralization tests are the most reliable assay for flavivirus antibody detection; however, these assays are not suitable for high-throughput processing due to their time-consuming and labor-intensive nature. In this study, we developed single-round infectious particles (SRIPs) with a luciferase gene for dengue virus types 1 to 4, Japanese encephalitis virus, and Zika virus for use in a safe, high-throughput neutralization assay. We performed neutralization tests against multiple flaviviruses using 147 serum samples that were collected from healthy donors residing in four distinct regions of Thailand in 2011 to 2012. The assay was useful for surveys of flavivirus seroprevalence. The data revealed that dengue virus type 1 (DENV-1) and DENV-2 were the predominant circulating flaviviruses in Thailand and that Zika virus has been circulating at a sustained level in Thailand since before 2012.

## INTRODUCTION

Arthropod-borne flaviviruses belonging to the genus *Flavivirus* in the family *Flaviviridae* are enveloped viruses with a positive-strand RNA genome of 9 to 12 kb that encodes three structural proteins (capsid, C; precursor-membrane, prM; and envelope, E) and seven nonstructural proteins (NS1, NS2a, NS2b, NS3, NS4a, NS4b, and NS5) (1). Approximately 40 flaviviruses exhibit virulence in humans (2), of which dengue virus types 1 to 4 (DENV-1 to -4), Japanese encephalitis virus (JEV), Zika virus (ZIKV), West Nile virus (WNV), yellow fever virus (YFV), and tick-borne encephalitis virus (TBEV) are particularly important because of the potential impact of their disease severity, global distribution, and number of patients. ZIKV disease was recently identified as an emerging infectious disease for which the World Health Organization (WHO) declared a public health emergency of international concern in 2016 (3).

Thailand is a hyperendemic country for flavivirus infections in Southeast Asia, where the annual average number of dengue infections was estimated to be approximately 67,000 cases from 1985 to 2009, with all four dengue serotypes cocirculating (4). In contrast, the prevalence of ZIKV in Thailand had not been extensively investigated before the 2015 outbreak in South America, where an association between ZIKV infection and microcephaly was reported. A recent serological survey provided evidence of the endemic ZIKV burden in Thailand (5). Although seven cases of ZIKV infection were reported in Thailand during 2012 to 2014 (6), the prevalence of ZIKV before 2012 has not been evaluated. The first epidemic of Japanese encephalitis (JE) occurred in the northern region of Thailand in 1969 (7). During the 1970s and 1980s, approximately 2,000 JE cases were reported annually (8). The Thai government has maintained a JE vaccination program in selected provinces since 1990, followed by the launch of a nationwide childhood JE vaccination program in 2000 (9). The Thai Ministry of Public Health revealed that the current JE vaccine coverage rate in 2- to 3-year-old children was approximately 90% (9). Recently, the annual number of JE patients in Thailand has been estimated to be 100 to 200 (10).

The reporting system for dengue surveillance in Thailand is well established, and epidemic data on dengue fever (DF), dengue hemorrhagic fever (DHF), and dengue shock syndrome (DSS) are disclosed to the public by the Thai Ministry of Public Health (https://ddc.moph.go.th/en/). However, syndromic surveillance is limited and may not accurately reflect the current epidemiological status of flavivirus prevalence, as most cases with flavivirus infection are asymptomatic or show a self-limited disease (11). Therefore, serological studies of healthy individuals are important for clarifying the prevalence of flaviviruses in endemic regions. Although several serology test kits are currently available for detecting flavivirus antibodies (e.g., enzyme-linked immunosorbent assay and immune chromatography), the cross-reactivity among flaviviruses sometimes makes it difficult to evaluate specific antibodies, especially in regions where two or more flaviviruses are in circulation. Neutralization tests are the most reliable serological assay and are capable of providing high specificity among flaviviruses (12). However, since functional neutralization tests require live virus as the assay antigen, performing neutralization tests is not suitable in laboratories that do not have access to live infectious viruses or which do not meet appropriate biosafety standards.

A novel neutralization assay system utilizing a flaviviral single-round infectious particles (SRIP) as an assay antigen for a conventional neutralization test has already been established (13–15). Briefly, an SRIP is a chimeric viral particle whose virion surface is constituted of the prM and E proteins of any flavivirus and which contains a subgenomic replicon derived from JEV (Nakayama strain) or DENV-1 (D1/Hu/Saitama/NIID100/2014 strain) (14, 16). Because the surface proteins (prM and E proteins) of flaviviruses are the targets of neutralizing antibodies (NAbs), SRIPs generated from a plasmid expressing the prM and E genes can be used in the neutralization test. The subgenomic replicon lacks coding regions for the prM and E proteins; thus, SRIP-infected cells do not generate progeny viruses in the supernatant, which represents a safety advantage (16). The nanoluciferase (NLuc) reporter gene is included in the replicon plasmid instead of the prM and E genes; thus, intracellular luciferase activity correlates with the number of infected cells in a high-throughput implementation (14). Furthermore, we previously demonstrated that luciferase activity was dose-dependently inhibited in the presence of NAb, demonstrating the utility of SRIPs in the conventional neutralization test (14).

In the present study, we established a yellow fever virus (YFV)-based SRIP production system corresponding to six flaviviruses (DENV-1 to -4, JEV, and ZIKV) and performed neutralization tests using stored serum samples collected from 147 healthy volunteers residing in four distinct regions in Thailand from 2011 to 2012 to investigate the seroprevalence of multiple flaviviruses in the country. We demonstrated that the flaviviral SRIP neutralization system could safely provide reliable serosurveillance results in a high-throughput implementation. The data revealed that DENV-1 and DENV-2 were the predominant circulating flaviviruses irrespective of region, whereas regions in which JEV was in circulation were limited. In addition, ZIKV was revealed to have been circulating at sustained levels since before 2012.

## RESULTS

### Generation of various flavivirus SRIPs with a yellow fever reporter replicon.

**FIG 1:**
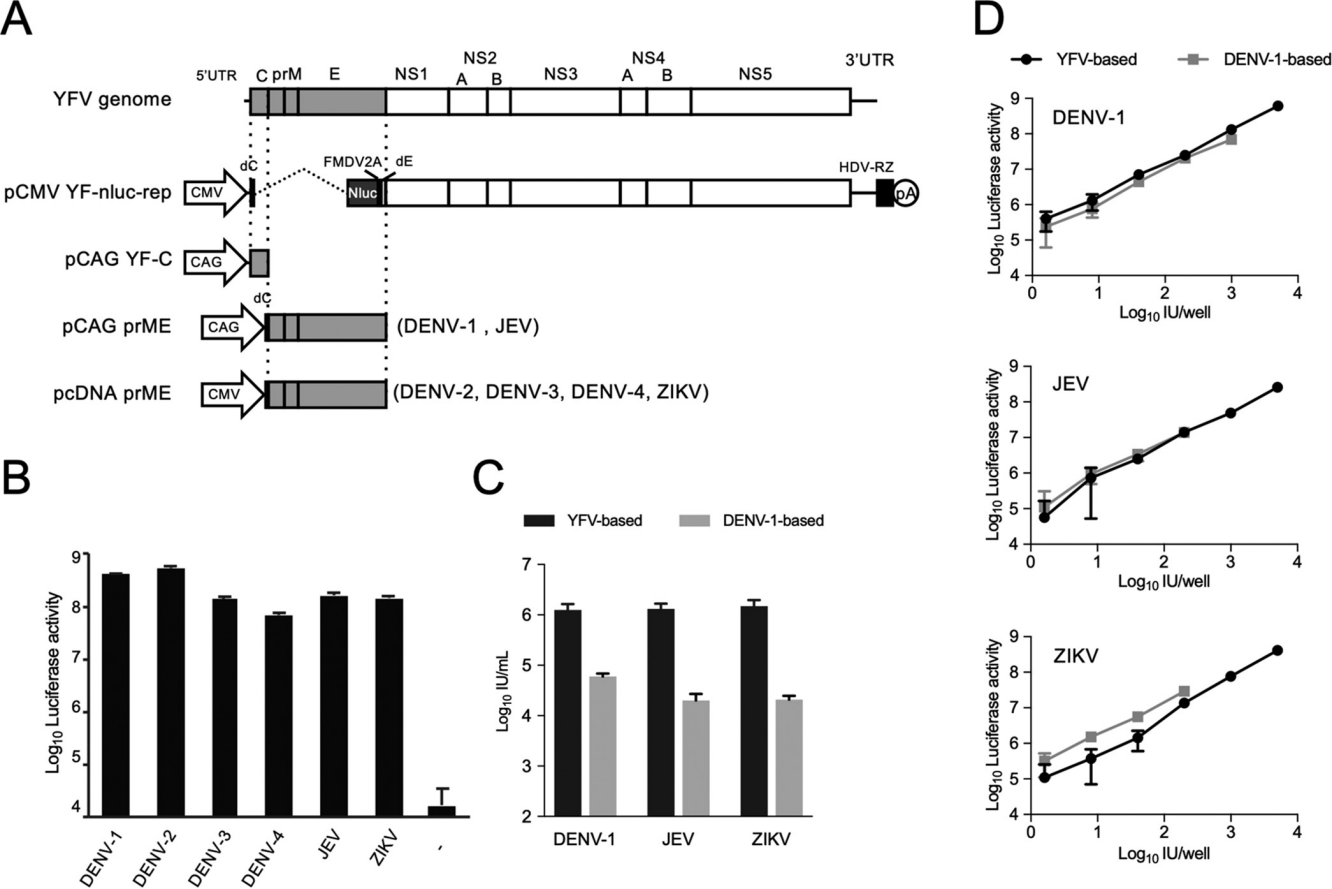
Construction of a DNA-based YFV replicon containing the nanoluciferase gene and generation of SRIPs for six flaviviruses. (A) Schematic representation of the YFV genome and replicon construct (pCMV YF-nluc-rep). Positions of the CMV promoter (CMV), nanoluciferase (Nluc) gene, 2A protein sequence of foot-and-mouth disease virus (FMDV2A), hepatitis delta virus ribozyme (HDV-RZ), polyadenylation signal (pA), and CAG promoter (CAG) are shown. Structural protein-expression plasmids were also constructed encoding YFV capsid (pCAG YF-C) or flavivirus prM/E genes derived from dengue virus types 1 to 4 (DENV-1 to -4), Japanese encephalitis virus (JEV), and Zika virus (ZIKV). One of the six plasmids expressing prM/E proteins was used to generate the corresponding SRIP. (B) Luciferase activity in Vero cells infected with SRIPs. Supernatants of 293T cells cotransfected with three plasmids (pCMV YF-nluc-rep, pCAG YF-C, and one of the prM/E-expressing plasmids) were harvested at 3 days posttransfection, and infectivity was determined in Vero cell monolayers based on luciferase activity 3 days after incubation. (C) Titers of SRIPs produced by transfection with YFV- or DENV-1-based replicon. YF-based (black bars) represents the SRIPs produced by transfection of 293T cells with pCMV YF-nluc-rep and pCAG YF-C. DENV-1-based (gray bars) represents the SRIPs produced by transfection of 293T cells with pCMV-D1-nluc-rep and pCAG-D1C. Each of the prME plasmids used for transfection is indicated. Dilutions of the supernatant collected at 3 days posttransfection were used to inoculate the monolayers of Vero cells. The cells were then fixed at 3 days postinfection and stained with anti-NS1 antibody, and the stained cells were then counted to determine the titers of the transfections. (D) Relationship between luciferase activity and the number of infected cells. Each SRIP (DENV-1, JEV, or ZIKV) generated with the YFV- or DENV-1-based replicon was serially diluted (5-fold dilutions) and used to inoculate Vero cells. Luciferase activity and inoculate IU/well were then plotted.

A DNA-based YFV replicon plasmid containing the NLuc gene instead of the partial C, prM, and E genes (pCMV YF-nluc-rep) and pCAG YF-C (expression plasmid for YFV mature C) were constructed (Fig. 1A) by following a previously described method (14). To generate the flavivirus SRIPs used as assay antigens for the high-throughput neutralization test, 293T cells were cotransfected with a mixture of three plasmids (pCMV YF-nluc-rep, pCAG YF-C, and one of the prME-expression plasmids derived from the following viruses: DENV-1, DENV-2, DENV-3, DENV-4, JEV, and ZIKV) (Fig. 1A). Sufficiently high luciferase activities were detected from Vero cells inoculated with supernatants containing SRIPs, compared to the control whose supernatant was harvested from cells transfected with pCMV YF-nluc-rep, pCAG YF-C, and an empty vector (–) instead of the prME-expression plasmid (Fig. 1B); these findings indicated that the SRIPs are suitable for functional assays. As shown in Fig. 1C, the SRIPs (DENV-1, JEV, and ZIKV) generated with the YFV-based replicon showed a high infectious titer compared to that observed with the DENV-1-based replicon that we reported previously (14). In addition, luciferase activity and the number of SRIP-infected cells were strongly correlated among different replicon systems (Fig. 1D). Therefore, we used the YFV-based replicon to produce SRIPs to further evaluate the neutralization titer of the samples.

### Sample classification.

**FIG 2:**
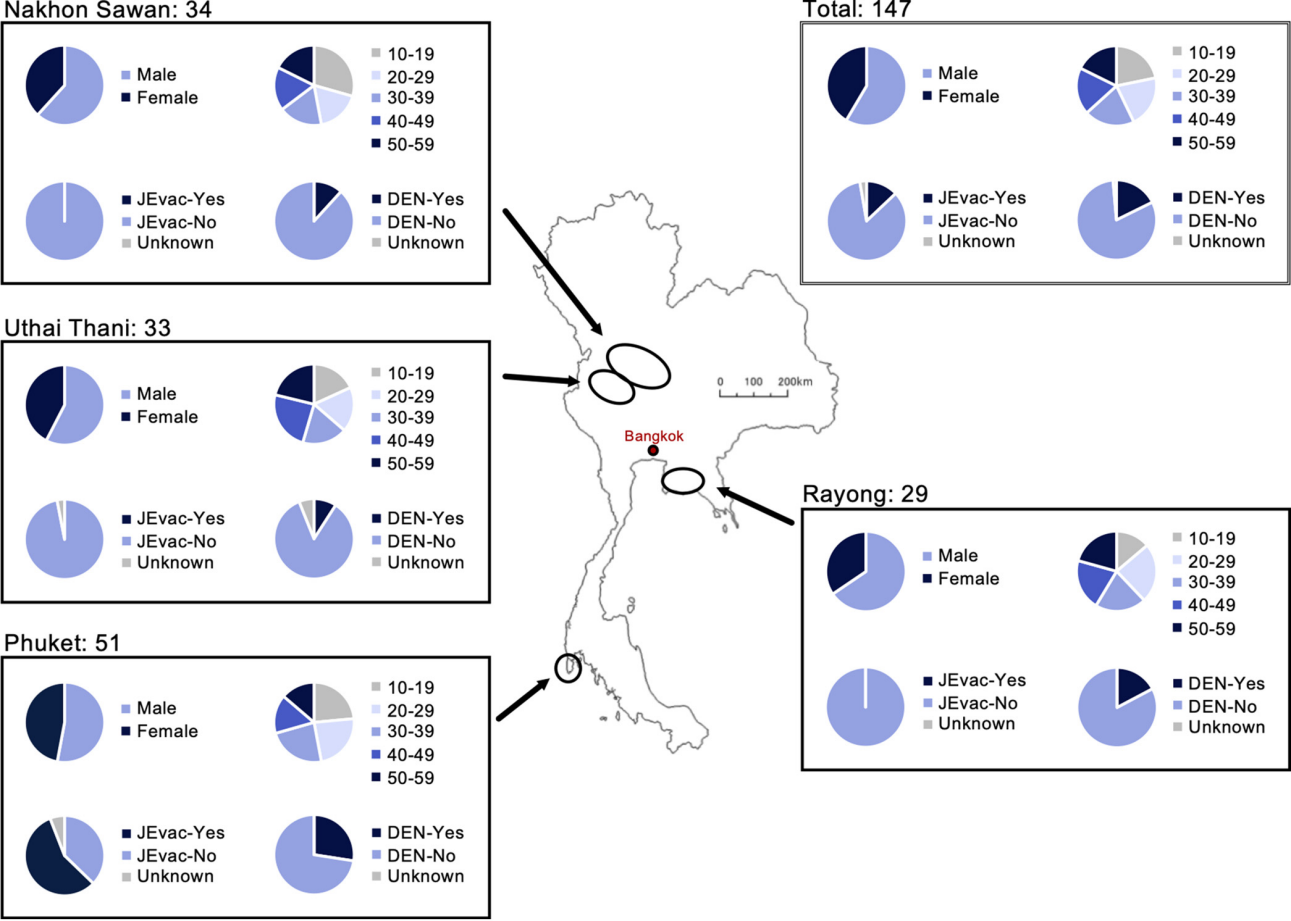
Map of Thailand showing the four provinces from which sera were collected. The proportions of sex, age, record of JE vaccination, and DENV infection history of the serum samples are shown for all regions (total) and individual regions.

A total of 147 serum samples were collected from healthy volunteers who provided written informed consent. Samples were collected from subjects in four provinces in Thailand (Nakhon Sawan [NS], Uthai Thani [UT], Rayoung [RY], and Phuket [PK]) from 2011 to 2012. NS and UT are located in the central-northern region. RY is located in the central region (approximately 200 km southeast of the Bangkok metropolis), and PK is an island in the south of the country (see map in Fig. 2). Based on the answers to questionnaires in the informed consent form, the serum samples were classified according to the following factors: (i) sex, (ii) age, (iii) record of JE vaccination, and (iv) history of DENV infection (Fig. 2). There were no significant differences in the subjects’ sex and age among the four regions. All subjects with a record of JE vaccination were located in PK. The proportion of subjects with a previous DENV infection history in PK (27.5%) was relatively high compared to those in NS (11.8%), UT (9.1%), and RY (17.2%).

### Pattern of neutralizing activity against flaviviruses in Thai populations.

**FIG 3:**
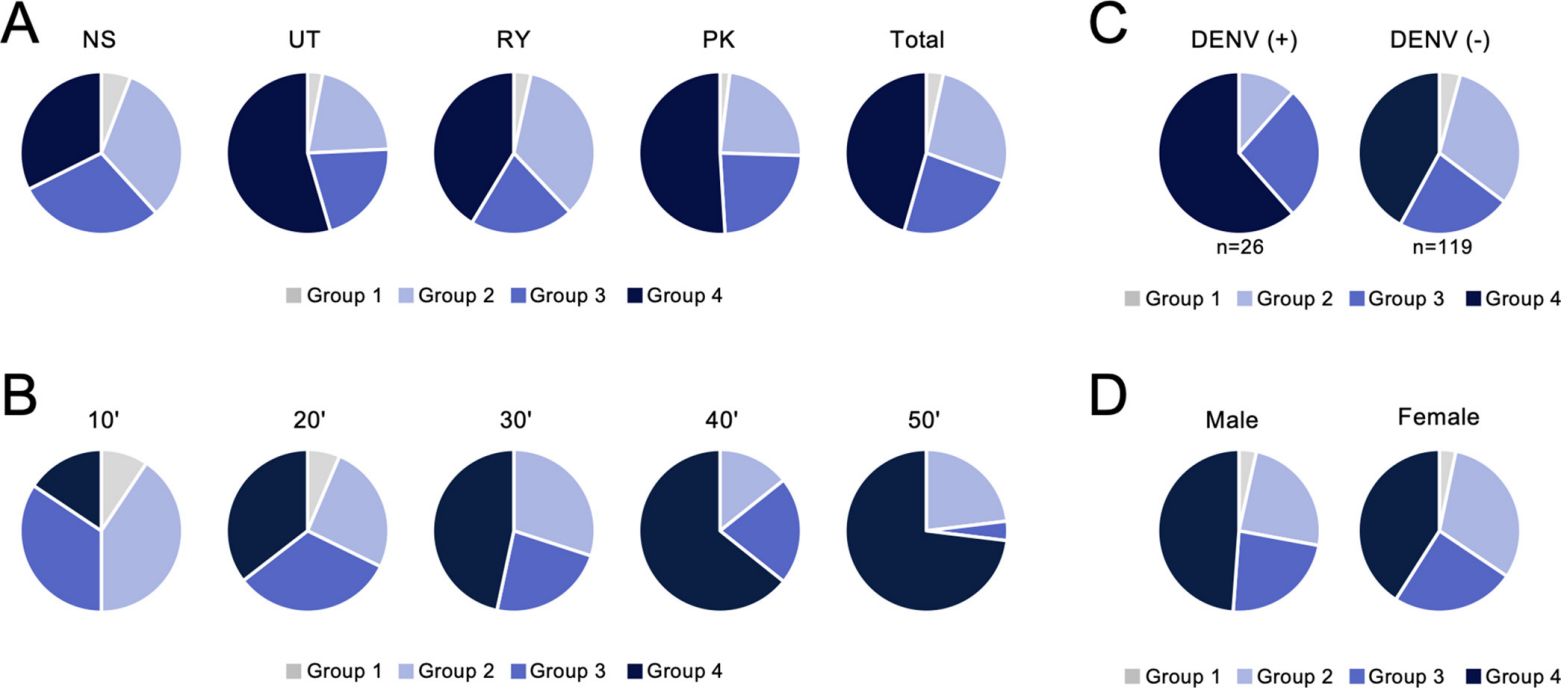
Flavivirus neutralizing activity patterns. Serum samples were classified into four groups according to neutralizing activity patterns (i.e., specific or showing cross-reactivities against the six flaviviruses). Group 1, IC_75_ titers against all six SRIPs were <1:10; group 2, IC_75_ titer against a single SRIP was 4-fold higher than those against the five other SRIPs; group 3, IC_75_ titers against any two SRIPs (among which there is a ≤4-fold difference) were 4-fold higher than against the four other SRIPs; group 4, none of groups 1 to 3 and represents substantial IC_75_ titers against three or more SRIPs, among which there was a <4-fold difference. (A to D) The proportions of each group are shown by (A) region, (B) age, (C) history of DENV infection, and (D) sex.

Neutralization tests using six flavivirus SRIPs (DENV-1, DENV-2, DENV-3, DENV-4, JEV, and ZIKV) were performed on 147 serum samples. NAb titers were obtained from the inhibitory concentration (serum dilution) that neutralized 75% of SRIP infection (IC_75_). Based on the IC_75_ titers and dose-dependent neutralizing activity patterns, the serum samples were classified into the following four groups: Group 1, IC_75_ titers against all six SRIPs were <1:10; group 2, IC_75_ titer against a single SRIP was 4-fold higher than those against the five other SRIPs; group 3, IC_75_ titers against any two SRIPs (among which there is a ≤4-fold difference) were 4-fold higher than those against the four other SRIPs; group 4, none of groups 1 to 3 and represents substantial IC_75_ titers against three or more SRIPs, among which there was a <4-fold difference. Representative dose-dependent neutralizing activity curves are shown in Fig. S1. There was no marked difference in the composition of the groups among the four regions, as shown in Fig. 3A. In contrast, large differences were observed in the age classification (Fig. 3B). Specifically, group 1 was composed exclusively of the 10s and 20s age groups. Although the total proportion of groups 2 and 3 comprised the majority (75.0%) of donors aged 10 to 19 years, these proportions decreased with advancing age. In contrast, group 4 comprised the majority (73.1%) of donors aged 50 to 59 years (Fig. 3B). These results indicate that the likelihood of having been infected with multiple flaviviruses tended to increase with age. In the classification based on DENV infection history, the total proportion of groups 3 and 4 among donors with a record of previous DENV infection (88.5%) was significantly higher (*P < *0.05) than those without previous infection (64.7%) (Fig. 3C). All participants in group 1 were classified in the group without DENV infection. There was no significant difference in compositions of male and female subjects within groups (Fig. 3D).

10.1128/mSphere.00339-21.1 FIG S1 Dose-dependent neutralizing activity curves showing typical patterns against flavivirus SRIPs for each group; (A) group 1, (B) group 2 (C) group 3, (D) group 4. These data were used to classify the samples into four groups and to calculate the IC_75_ NAb titers shown in Fig. 3, 5, 6, and 7A. Download FIG S1, PDF file, 0.04 MB. Copyright © 2021 Yamanaka et al. 2021 Yamanaka et al. https://creativecommons.org/licenses/by/4.0/ This content is distributed under the terms of the Creative Commons Attribution 4.0 International license.

### Prevalence of NAb against flavivirus showing the highest titer.

**FIG 4:**
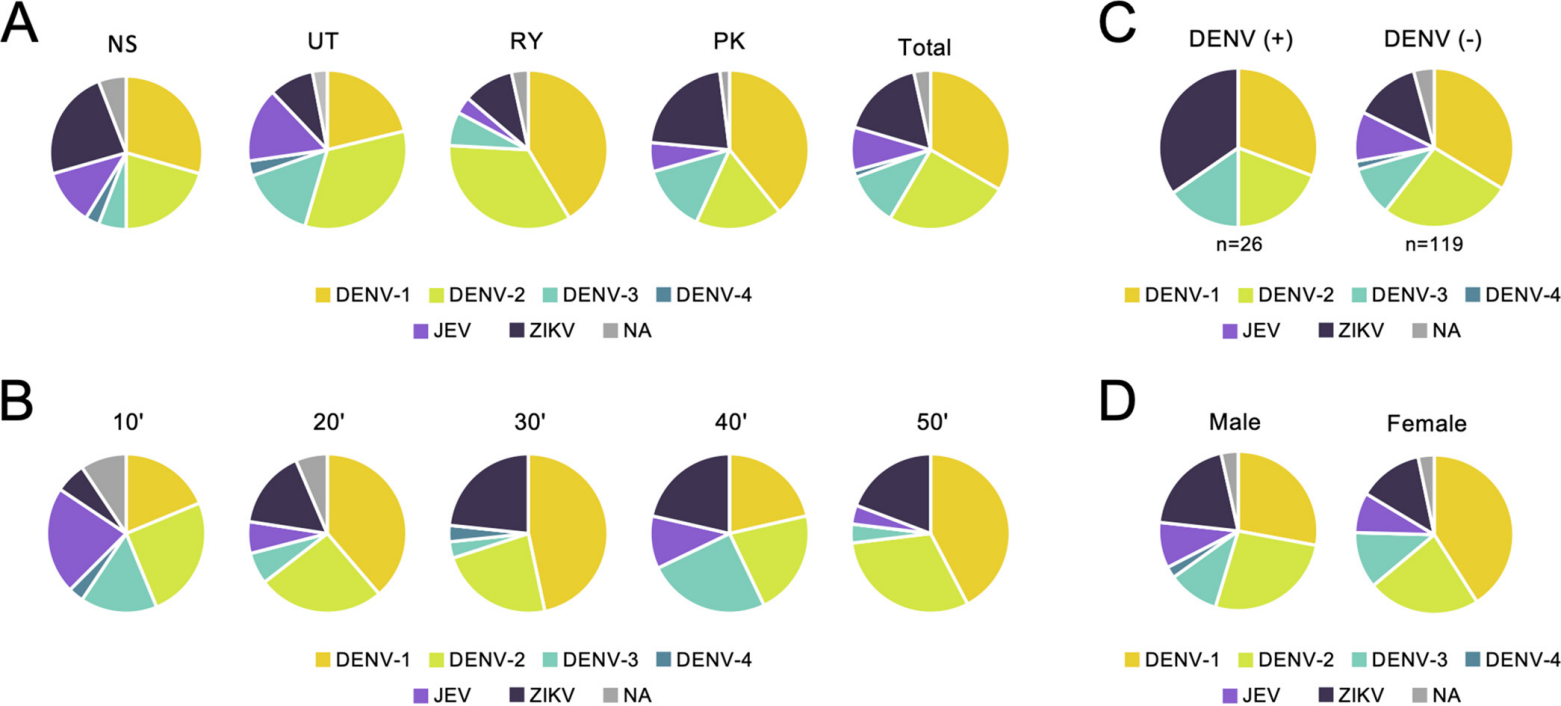
Prevalence of antibodies showing the highest neutralizing activity among flaviviruses. (A to D) The prevalence of NAbs with the highest titer among six flavivirus SRIPs is shown according to each condition; (A) region, (B) age (C) history, of DENV infection, and (D) sex. NA indicates populations with IC_75_ titers of ≤1:10 against all six SRIPs.

Figure 3A shows that approximately 70% of the serum samples analyzed showed neutralizing activity against multiple flaviviruses (groups 3 and 4), which would make it difficult to correctly identify the previous infecting viruses, even though the neutralization test is considered to be a gold standard assay. Therefore, to clarify the primary flavivirus infection, the prevalence of NAbs showing the highest titer in each individual was summarized by region, age, DENV infection history, and sex (Fig. 4A to D). Similar trends in antibody prevalence were observed irrespective of region. Notably, the majority of the populations showed the highest NAb titer against DENV-1 or DENV-2 in RY (75.9%), followed by PK (56.9%), UT (54.5%), and NS (50.0%) (Fig. 4A), suggesting that DENV-1 and DENV-2 are historically predominant in Thailand. Interestingly, ZIKV had the third highest prevalence in a total of the four regions (17.0%), with prevalence being particularly high in NS (23.5%) and PK (21.6%). Participants showing the highest titer against DENV-4 were the minority in Thailand (1.4%). Populations showing the highest NAb titer against JEV were observed in NS (11.8%) and UT (15.2%), while the prevalence in RY and PK were 3.4% and 5.9%, respectively. Fig. 4B shows that the prevalence of JE antibodies was highest in the 10’s age group (21.9%), among which only two of seven individuals had a record of JE vaccination, suggesting that the other five individuals might have experienced a JEV infection. Furthermore, it should be noted that the prevalence of ZIKV NAb in populations with a DENV infection history (34.6%) was greater than the prevalence of individual DENV NAbs (Fig. 4C). Similar patterns were observed between male and female subjects (Fig. 4D).

### Individual IC_75_ data against six flavivirus SRIPs.

**FIG 5:**
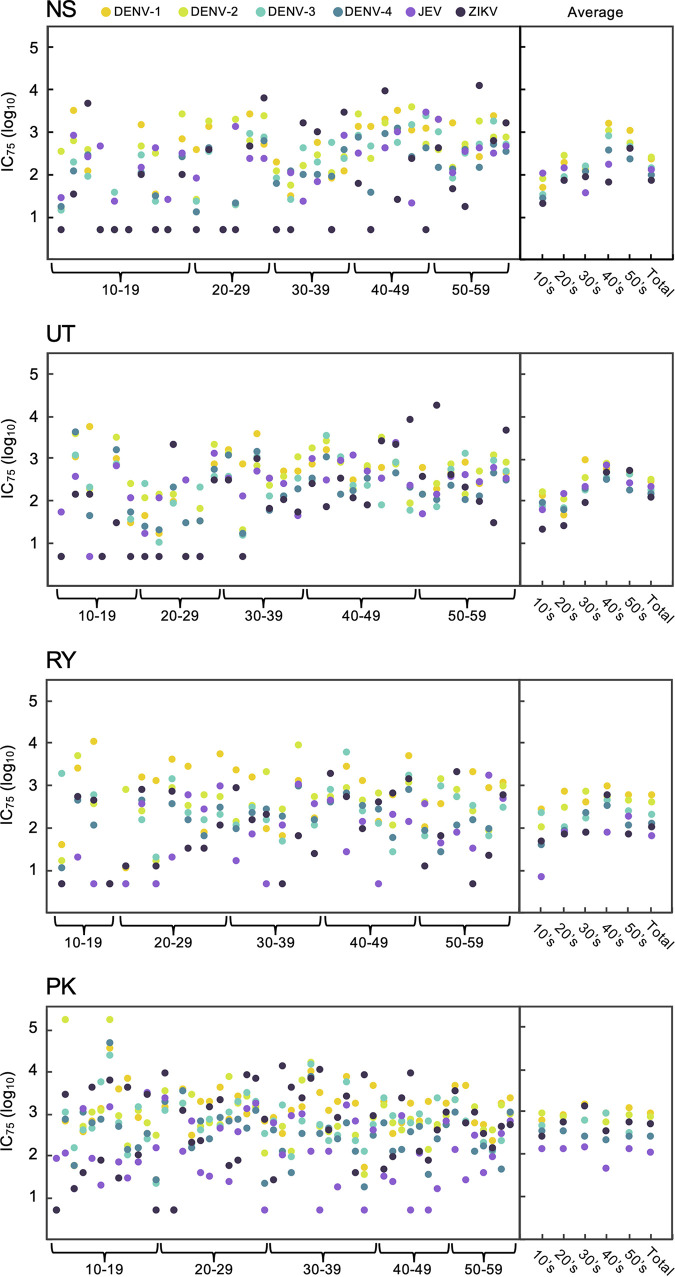
Individual IC_75_ data against six flavivirus SRIPs. The dots in the vertical direction represent individual IC_75_ titers against DENV-1, DENV-2, DENV-3, DENV-4, JEV, or ZIKV in a single serum sample. The *x* axis indicates the range of ages in ascending order. Averages in the 10 to 19, 20 to 29, 30 to 39, 40 to 49, and 50 to 59 age groups and the total are shown to the right of each panel. For cases in which the IC_75_ titer was lower than 1:10, the titer was defined as 1:5.

**FIG 6:**
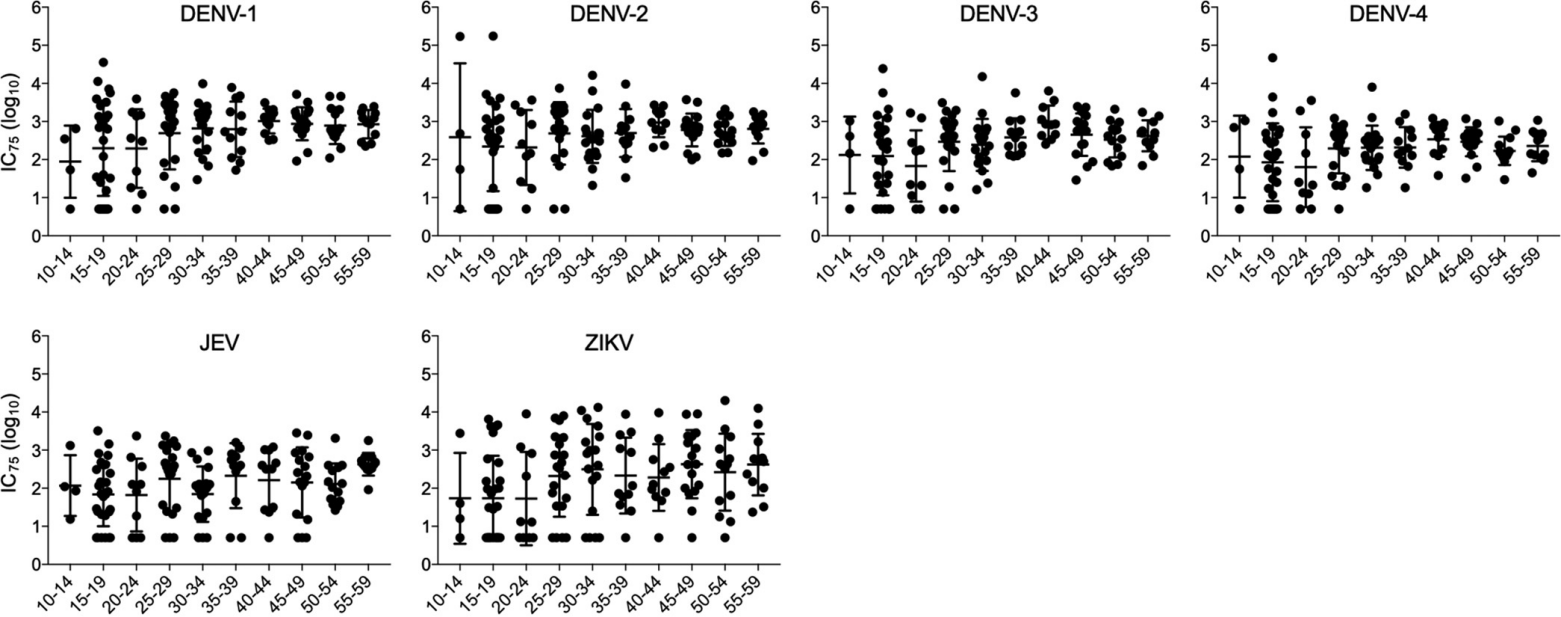
Individual seroprevalence of flavivirus SRIP antigens. The column scatterplots represent IC_75_ values against each of the six SRIPs (*y* axis) according to age groups (*x* axis). For cases in which the IC_75_ titer was <1:10, the titer was defined as 1:5.

Figure 5 shows individual and average IC_75_ titers against the six flavivirus SRIPs in the serum samples from each region. Average IC_75_ titers against DENV-1 or DENV-2 were consistently higher than the other flaviviruses in all four regions, with a minor exception. Specifically, the highest average IC_75_ against JEV was observed in certain age groups (10s in NS and 20s in UT) despite the absence of a record of JE vaccination. Interestingly, most of the population over 30 years old in UT, RY, and PK had IC_75_ titers of ≥1:10 against ZIKV. Although several participants over 30 years old in NS had IC_75_ titers of <1:10 against ZIKV, the seroprevalence (23.5%) against ZIKV in NS was the highest among the four regions (Fig. 4A). These results suggested that ZIKV was endemic at different times in each region of Thailand. Individual IC_75_ titers were also plotted for each SRIP (Fig. 6). The data clearly showed that seronegative samples (<1:10) against each virus decreased with advancing age. Notably, total populations >30 years old had IC_75_ titers of ≥1:10 against all four DENV serotypes. In contrast, several participants >30 years old were seronegative against JEV and ZIKV.

### JE vaccine records.

**FIG 7:**
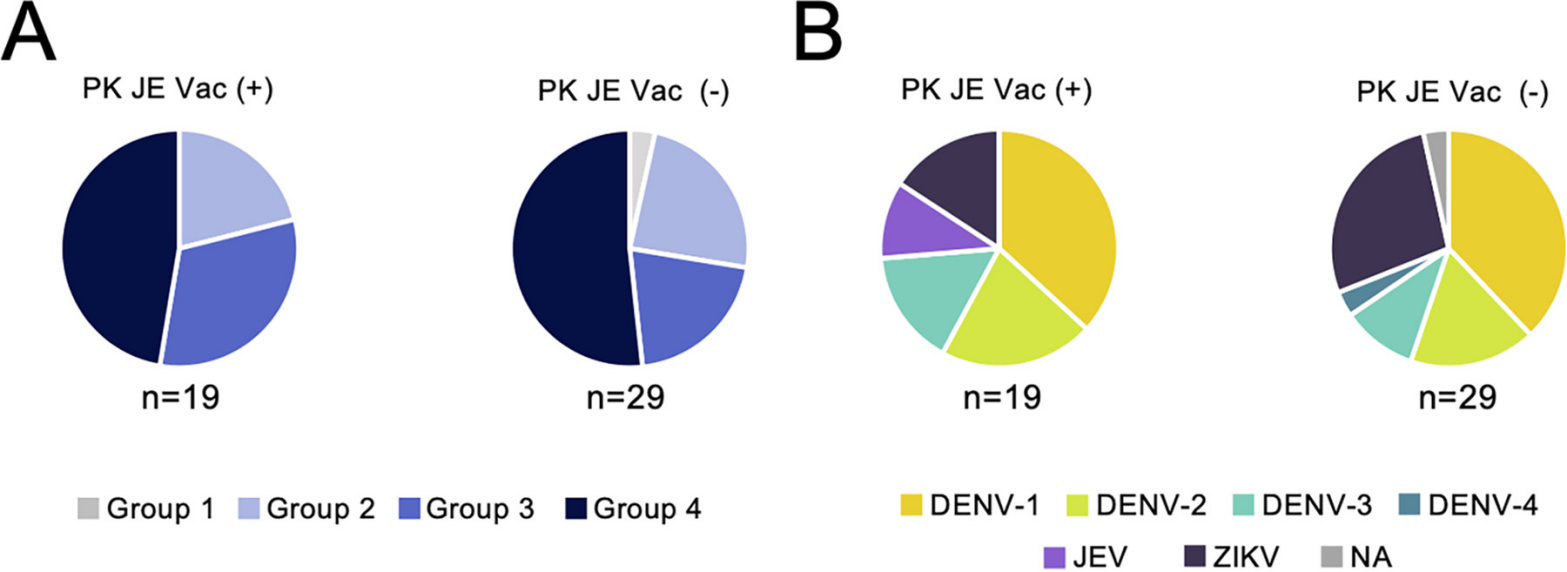
Influence of JE vaccination upon neutralizing activity patterns and prevalence of the highest NAb among flaviviruses. A total of 48 samples collected from PK were used to investigate whether JE vaccination affected antibody reactivity against six flaviviruses. Three samples were excluded because of missing JE vaccination information. (A) Effect on neutralizing activity pattern. Samples were classified by neutralizing activity patterns (i.e., specific or showing cross-reactivity) as shown in Fig. 3. (B) Effect on NAb titer. The prevalence of NAbs with the highest titer among flaviviruses is shown.

We analyzed whether previous JE vaccination affected the neutralization activity against the six flavivirus SRIPs using serum samples collected from PK, where 29 of 48 participants had a record of vaccination. There were no obvious differences between JE vaccine recipients and nonrecipients in the classification based on the neutralizing activity patterns (Fig. 7A) or in the classification based on the highest NAb titers against each flavivirus (Fig. 7B).

## DISCUSSION

The present serology study of flaviviruses in Thailand used serum samples collected from healthy donors residing in four distinct geographical regions. The primary advantage of this study is the use of six flavivirus SRIPs as antigens for the conventional neutralization test, which is the most reliable assay for antibody surveys and is considered the gold standard. We demonstrated that the present SRIP neutralization system is a suitable replacement for the conventional plaque reduction neutralization test. Since the SRIP neutralization system does not require live viruses, it enables the performance of neutralization tests against a broad range of flaviviruses in laboratories lacking sufficient biohazard control measures. Moreover, the SRIP system can circumvent difficulties associated with obtaining foreign flavivirus strains. Since the YFV-based replicon can produce higher titers of SRIPs for different flaviviruses than the DENV-1-based replicon (Fig. 1C), the system described in this study could be applied to numerous flaviviruses.

Serological surveys focusing on elucidating the historical status of flavivirus in endemic countries is important for public health. Thailand is a DENV hyperendemic country in which the predominant serotypes and genotypes have shifted frequently (17). The current findings showed that DENV-1 and DENV-2 were the predominant serotypes in Thailand, irrespective of region, which was consistent with the findings of a previous study (18). Individual data of IC_75_ titers against each SRIP revealed that all samples from subjects >30 years of age had NAbs (≥1:10) against all four DENV serotypes (Fig. 6). These results suggest that most Thai people may be infected with multiple serotypes of DENV during the first decades after birth, which may induce cross-neutralization among the four DENV serotypes. In contrast, some individuals over the age of 30 were seronegative for JEV and ZIKA, implying that the introduction of the JE vaccine program has reduced the circulation of JEV. In addition, ZIKV is considered to have been introduced into Thailand relatively recently, and/or it may be less prevalent than DENVs. Interestingly, some participants possessed higher NAb titers against JEV and/or ZIKV than titers against DENVs. Specifically, a relatively high proportion of participants with the highest NAb titer against ZIKV was observed in NS and PK, as well as populations aged 20 to 59 years old (Fig. 4A and B). These findings suggest that ZIKV, which causes Zika fever in humans and is considered to be a recent emerging infectious disease, has already been circulating extensively in limited regions of Thailand since before 2012. Furthermore, among the six antigens, a high proportion of participants with dengue infection history had the highest NAb titer against ZIKV (Fig. 4C). Because of the considerable structural and genetic similarities between ZIKV and DENV, the antibodies induced by infection with these viruses could be a cross-reactivity concern in serological assays. However, recent reports have shown that neutralizing antibody titers can be used to distinguish between Zika and dengue infections when both viruses are assayed (14, 19). When considering individual data, NAb titers against ZIKV were 20-fold higher than those against DENVs in some participants, and vice versa (Fig. 5). In addition, among the 25 individuals whose sera showed the highest neutralizing antibody titers against ZIKV in this study, 10 individuals (40%) displayed at least a 4-fold difference between neutralizing antibody titers against ZIKV and other flaviviruses, suggesting that cross-reactive neutralization between DENV and ZIKV was limited. Therefore, individuals with a history of dengue infection could have been infected with ZIKV but were diagnosed with dengue due to the similarity in their symptoms and the use of a single seroassay for dengue.

Relatively high proportions of participants with the highest NAb titers against JEV were observed in UT and NS, as well as populations aged 10 to 19 years old (Fig. 4A and B), suggesting that JEV may have been in circulation in specific regions and in younger subjects. These results are consistent with previous reports of more severe JE epidemics in the northern regions (7, 20), whereas JEV prevalence is lower than that of DENV.

The majority (40.6%) of participants aged 10 to 19 years old were classified into group 2, which was defined as having a single infection history (Fig. 3). Moreover, group 1 (participants who are considered to have no infection history) was found only in subjects aged 10 to 29 years old. In contrast, the majority of subjects aged >30 years were classified in group 4, which was defined as having a history of multiple infections. These results suggest that the likelihood of exposure to flaviviruses in the wild increases with advancing age. Therefore, it is speculated that children younger than 10 years of age would be classified mainly into group 1 or 2.

Usage of Dengvaxia, the first FDA-approved dengue vaccine from Sanofi Pasteur, is currently restricted to only seropositive individuals who have previously been infected with DENVs, since this vaccine may increase disease severity in seronegative individuals when they are subsequently infected with DENVs (21, 22). Based on our serological results, all participants in group 1 are strongly recommended not to get vaccinated with Dengvaxia. In contrast, individuals in groups 3 and 4 (and some participants in group 2) may receive the vaccination if they are found to be seropositive for DENVs. Although our SRIP neutralization system is not an instant diagnostic test, the detection of functional antibodies using DENV SRIPs is considered to be important for vaccine recipients, as it provides a reliable serostatus.

JE vaccine history did not affect cross-reactive neutralization patterns or the highest NAb titer (Fig. 7A and B). This indicates that the JE vaccine is not considered to induce cross-reactive NAbs against other flaviviruses and that it does not confer protection against other flavivirus infections.

In conclusion, in an analysis of 147 Thai serum samples, we demonstrated the application of our novel high-throughput neutralization system using six flavivirus SRIPs (DENV-1, DENV-2, DENV-3, DENV-4, JEV, and ZIKV), which is simpler, more rapid, and safer than conventional neutralization tests. We found that antibody prevalence against DENV was very similar in the geographical regions studied, whereas the prevalence of NAbs against ZIKV and JEV varied depending on region, age, and history of DENV infection. Moreover, our results strongly suggest that ZIKV has been circulating at a sustained level in Thailand since before 2012. The present survey of antibody prevalence may provide justification for an immediate introduction of the dengue vaccine in parts of Thailand.

## MATERIALS AND METHODS

### Serum samples.

Serum samples were collected from 147 healthy volunteers aged between 10 and 59 years old from October 2011 to October 2012 residing in four provinces of Thailand, Nakhon Sawan (*n* = 34), Uthai Thani (*n* = 33), Rayoung (*n* = 29), and Phuket (*n* = 51). This study was approved by the research ethics institutional review board of Mahidol University (approval no. MUTM 2017-083-01) and the National Institute of Infectious Diseases (approval no. 898).

### Plasmid construction.

A YFV subgenomic replicon plasmid was generated using cDNA from the YFV 17D vaccine strain. Briefly, fragments of the 5′ untranslated region (UTR), 21 amino acids (aa) of the N-terminal C-coding region, the NLuc gene, the foot-and-mouth disease virus (FMDV)-2A gene, 24 aa of the C-terminal E protein transmembrane sequence, following the NS1-NS5 coding sequence and the 3′-UTR sequence from YFV 17D were cloned between the cytomegalovirus (CMV) promoter and the hepatitis delta virus ribozyme (HDV-RZ) from pCMV-D1-nluc-rep (14), as shown in Fig. 1. To generate the YFV capsid expression plasmid pCAG YF-C, a 100-aa cDNA sequence encoding the mature capsid was amplified from a cDNA used for amplification of the replicon. The resultant fragments were cloned into pCAGGS.

To generate the DENV-1 prME expression plasmid pCAG-D1-YG1prME, cDNA encoding the prME of DENV-1 (D1/Hu/Saitama/NIID100/2014) (14) was amplified and cloned into pCAGGS. Plasmids pcD2ME, pcD3ME^SG^, pcD4ME^Th^, pCAG-JEprME, and pcZIKME are expression plasmids for the signal, prM, and E of DENV-2 (D2-New Guinea C), DENV-3 (D3/SG/05K4454DK1/2005), DENV-4 (D4-ThD4_0476_97), JEV (Nakayama), and ZIKV (MR766-NIID), respectively, as described previously (14, 16).

### Cells and reagents.

Human embryonic kidney 293T cells and African green monkey kidney Vero cells were maintained in Dulbecco’s modified Eagle’s medium (Wako Pure Chemical Industries) supplemented with 10% fetal bovine serum (FBS). All cell lines were cultured at 37°C in a 5% CO_2_ incubator.

### Production of each SRIP.

293T cells were grown in a 10-cm cell culture dish and cotransfected with three plasmids—2.5 μg of replicon plasmid (pCMV YF-nluc-rep), 1.25 μg of capsid-expression (pCAG YF-C) plasmid, and 1.25 μg of individual prME-expression plasmids—using Lipofectamine LTX and Plus reagent (Invitrogen). On day 2 after cotransfection, the culture supernatant was replaced with fresh medium supplemented with 10 mM HEPES buffer. The medium was harvested on day 3 after the transfection and filtered through a 0.22-μm syringe filter; this medium was used as the SRIP antigen in neutralization tests using Vero cells. Production of SRIPs using the DENV-1-based replicon plasmid (pCMV-D1-nluc-rep) and capsid plasmid (pCAG-D1C) was performed as described previously (16).

### Titration of SRIPs.

To determine the infectious titer of SRIPs, Vero cells were plated in multiwell plates and infected with serially diluted SRIPs. After incubating the plate at 37°C for 5 to 6 h, the medium was replaced and the plate was incubated for 3 days at 37°C. Following incubation, monolayers were rinsed with phosphate-buffered saline (PBS), fixed in cold acetone/methanol (1:1), and then blocked with a nonfat milk solution (Block Ace; DS Pharma Biomedical) for 30 min at room temperature. Samples were then incubated with mouse anti-DENV NS1 antibody (ab214337; Abcam) for the DENV replicon or anti-YFV NS1 (BF-077; BioFront) for the YFV replicon for 60 min at room temperature, followed by Alexa Fluor 488-labeled goat anti-mouse IgG secondary antibody (Invitrogen). Infectious units (IU) per volume were calculated by counting stained cells.

### Luciferase activity reduction neutralization test.

SRIP antigens were adjusted to 50 to 100 infectious units/well for the neutralization assay, as reported previously (14). Then, 4-fold serially diluted serum samples were mixed with SRIP antigens at a 1:1 ratio. After incubating the mixtures at 37°C for 2 h, each serum-SRIP mixture was inoculated onto Vero cell monolayers in 96-well tissue culture plates. After incubating the plate at 37°C for 2 h, extra culture medium was added, and the plate was incubated at 37°C for another 3 days. After incubation, the Vero cells were washed once with PBS and lysed with passive lysis buffer (Promega). Luciferase activity was subsequently determined using the Nano-Glo luciferase assay system (Promega). Each sample was assayed in triplicate. The neutralization titers were expressed as the serum dilution producing 75% inhibition (IC_75_) and compared with luciferase activity obtained using a no-serum control, which was calculated by the FORECAST function in Microsoft Excel (Microsoft Corporation).
