# Supplementary figures and images for: Seroprevalence of Flavivirus Neutralizing Antibodies in Thailand by High-Throughput Neutralization Assay: Endemic Circulation of Zika Virus before 2012
Source: mSphere. 2021 Jul 14;6(4):e00339-21. doi: 10.1128/mSphere.00339-21 (PMC8386448; doi:10.1128/mSphere.00339-21)

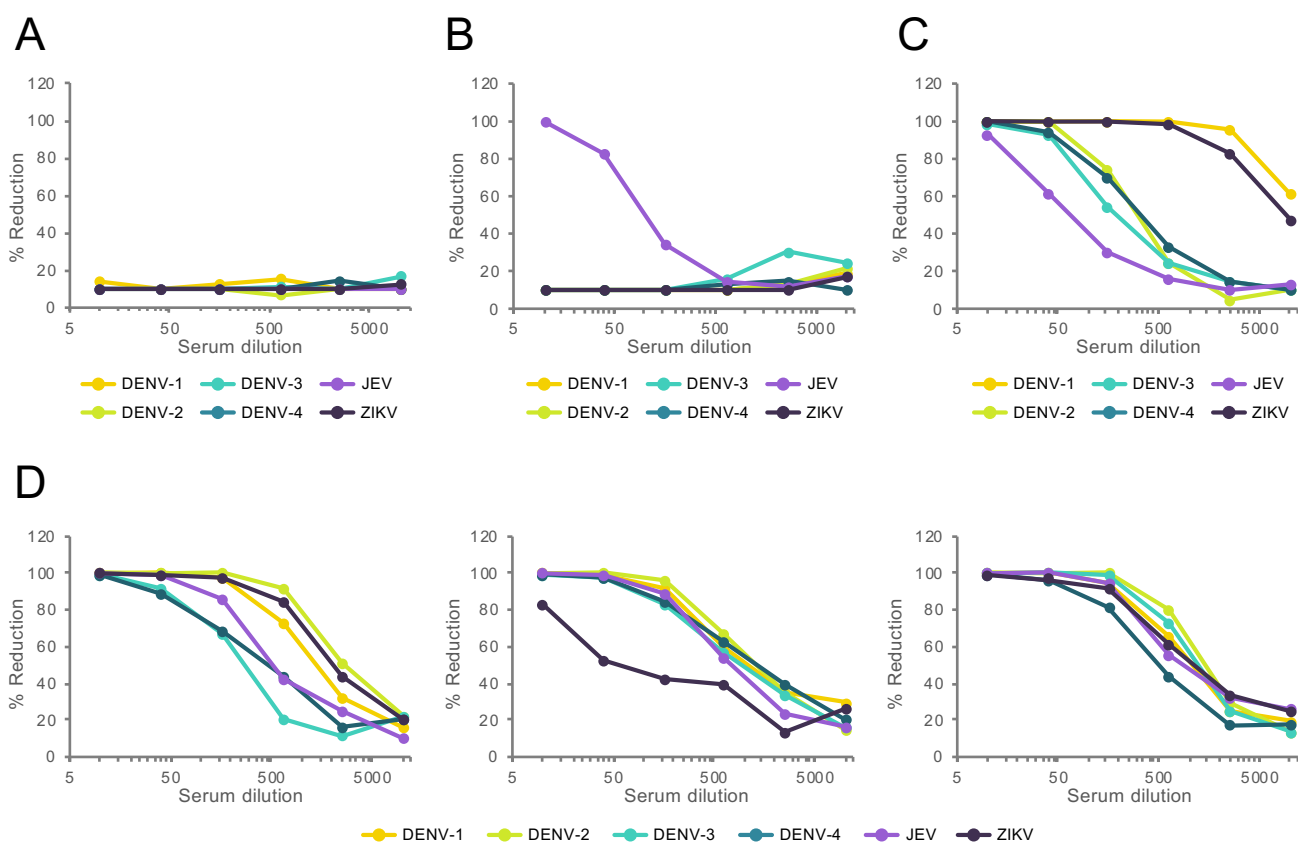

Supplement: FIG S1 [file msphere.00339-21-sf001.pdf]
